# Alternative moth-eye nanostructures: antireflective properties and composition of dimpled corneal nanocoatings in silk-moth ancestors

**DOI:** 10.1186/s12951-017-0297-y

**Published:** 2017-09-06

**Authors:** Mikhail Kryuchkov, Jannis Lehmann, Jakob Schaab, Vsevolod Cherepanov, Artem Blagodatski, Manfred Fiebig, Vladimir L. Katanaev

**Affiliations:** 1 Department of Pharmacology and Toxicology, University of Lausanne, Rue du Bugnon 27, 1011 Lausanne, Switzerland; 2 Department of Materials, ETH Zurich, Vladimir-Prelog-Weg 1-5/10, 8093 Zurich, Switzerland; 3 School of Biomedicine, Far Eastern Federal University, Sukhanova Street 8, Vladivostok, 690922 Russian Federation

**Keywords:** Moth-eye structures, Antireflective nanocoatings, Biomimetic materials, Silkmoth

## Abstract

Moth-eye nanostructures are a well-known example of biological antireflective surfaces formed by pseudoregular arrays of nipples and are often used as a template for biomimetic materials. Here, we provide morphological characterization of corneal nanostructures of moths from the *Bombycidae* family, including strains of domesticated *Bombyx mori* silk-moth, its wild ancestor *Bombyx mandarina*, and a more distantly related *Apatelodes torrefacta*. We find high diversification of the nanostructures and strong antireflective properties they provide. Curiously, the nano-dimple pattern of *B. mandarina* is found to reduce reflectance as efficiently as the nanopillars of *A. torrefacta*. Access to genome sequence of *Bombyx* further permitted us to pinpoint corneal proteins, likely contributing to formation of the antireflective nanocoatings. These findings open the door to bioengineering of nanostructures with novel properties, as well as invite industry to expand traditional moth-eye nanocoatings with the alternative ones described here.

**Electronic supplementary material:**

The online version of this article (doi:10.1186/s12951-017-0297-y) contains supplementary material, which is available to authorized users.

## Introduction

Moth-eye nanostructures—pseudo-regular arrays of nanopillars first described on corneal surfaces of the nocturnal moth *Spodoptera eridania* [1]—served as a paradigm for bio-inspired antireflective coating with broad technological applications, from solar cells to art paintings [2–5]. Gradually matching the refractive index of air to that of the lens material, these nanostructures minimize light reflectance and maximize perception. While physics of the anti-reflectance by nanopillar arrays is well-understood, permitting reliable simulations [6, 7], nature also designed other types of corneal nanocoatings [8], of which some were shown to play the antireflective function too [9, 10]. Numerous studies on artificial nanocoatings showed that the shape, dimensions, as well as the packing order of the nanostructures contribute to the anti-reflectivity [11–14].

Model insect organisms, permitting genetic manipulations and/or providing complete information on their genome sequences, have been instrumental in advancing the research on insect molecular, developmental, and cell biology [15]. Following our research on the corneal nanocoatings in *Drosophila melanogaster* [16, 17], we are now turning to another famous model insect—the silkmoth *Bombyx mori*.

More than 1000 different *B. mori* silkworm strains exist worldwide, having different phenotypic and genomic features [18, 19]. The silkworm was domesticated in East Asia from wild *B. mandarina* moths some 5000 years ago, losing several features essential in the wild habitat on the expense of maximizing silk production [20]. *Bombyx* moths’ genomes have been fully sequenced [18, 19], increasing their importance as genetic model organisms. While many aspects of the *Bombyx* biology have been analyzed, these insects have not been previously studied in terms of their corneal morphology and properties. We hypothesized that distinct corneal nanocoatings may be found in the wild vs. domesticated silkmoths.

## Results and discussion

**Fig. 1:**
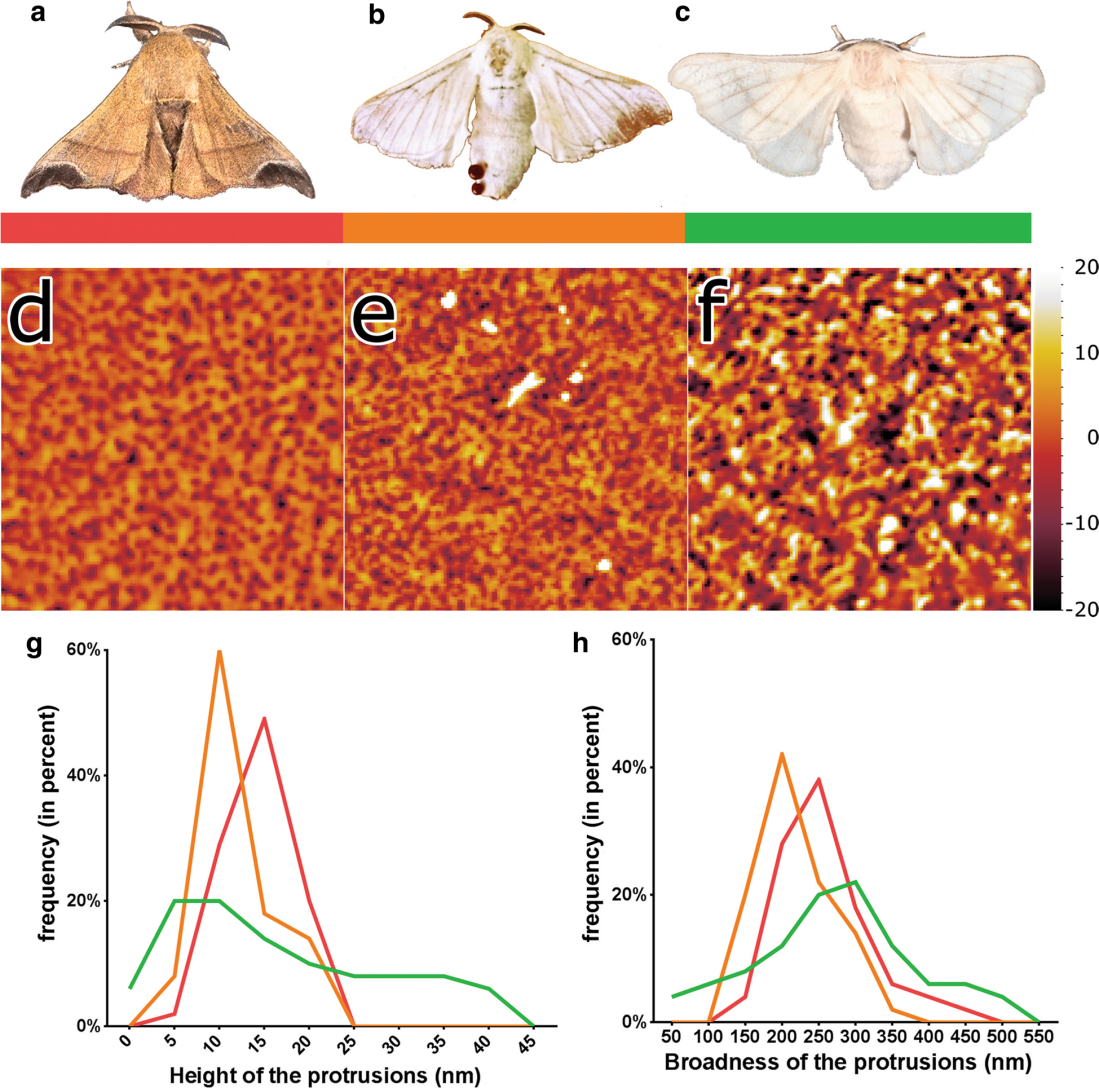
Corneal nanocoatings in *Bombyx* moths. **a**–**c** Photographs of *B. mandarina* (**a**), *B. mori* from Vietnam (**b**) and *B. mori* from Japan (**c**). **d**–**f** Representative AFM scans (5 × 5 µm) of corneal surfaces of the *Bombyx* species presented in **a**–**c**. The height dimension of the surface (in nm) is indicated by the *color scale* next to (**f**) with the mean set to zero. **g**, **h** Calculation of the height of protrusions (from the lowest point up to the next highest point (**g**) and their broadness (**h**) of *B. mandarina* (in *red*), *B. mori* [Vn] (in *orange*), and *B. mori* [Jp] (in *green*); n = 50

To explore differences between corneal nanocoatings in wild and domestic silkmoths, an atomic-force microscopy (AFM) analysis has been performed, comparing the corneal surfaces of *B. mandarina*, to the samples from two different *B. mori* strains obtained from Japan (Jp) and Vietnam (Vn, see “Materials and methods”; Fig. 1a–c). Unusually for Lepidopterans, different species of which so far have displayed different varieties of nanopillars, in some species fused into mazes or parallel strands [8], corneal surfaces of *B. mandarina* reveal a clear nano-dimpled pattern (Fig. 1d), previously described in insects of other orders, such as earwigs or *Carabidae* beetles [8]. Curiously, corneae of the two *B. mori* strains reveal different degrees of corruption of this pattern, with the Vn strain depicting a dimple-to-maze transition previously seen in other insects (e.g. bug from *Pyrrhocoridae* family [8]), and the Jp strain—a complete degeneration into sporadic irregularities (Fig. 1e–h). Fourier analysis [16, 21, 22] confirms this visual inspection, indicating that the *B. mandarina* and *B. mori* [Vn] corneal structures are close to quasi-random, while the *B. mori* [Jp]—to completely random structures (Additional file 1: Figure S1). Fully random structures are known to show less anti-reflectance than the quasi-random structures [11, 12], such as those we see in *B. mandarina* and *B. mori* [Vn] (Additional file 1: Figure S1). As *B. mori* [Jp] corneae further possess randomization in the broadness and depth of the nanostructures (Fig. 1), we may expect even stronger loss of the anti-reflectivity. Using finite-difference time domain-based approximations of the Maxwell function, simulations of the reflectance pattern of structured surfaces were found to match the results received in the process of the optical experiments [6, 7, 23, 24]. However, such simulations in general do not predict antireflective properties of nanostructures, whose dimensions are below 30–50 nm [7, 25], such as those we see in the *Bombyx* species.

**Fig. 2:**
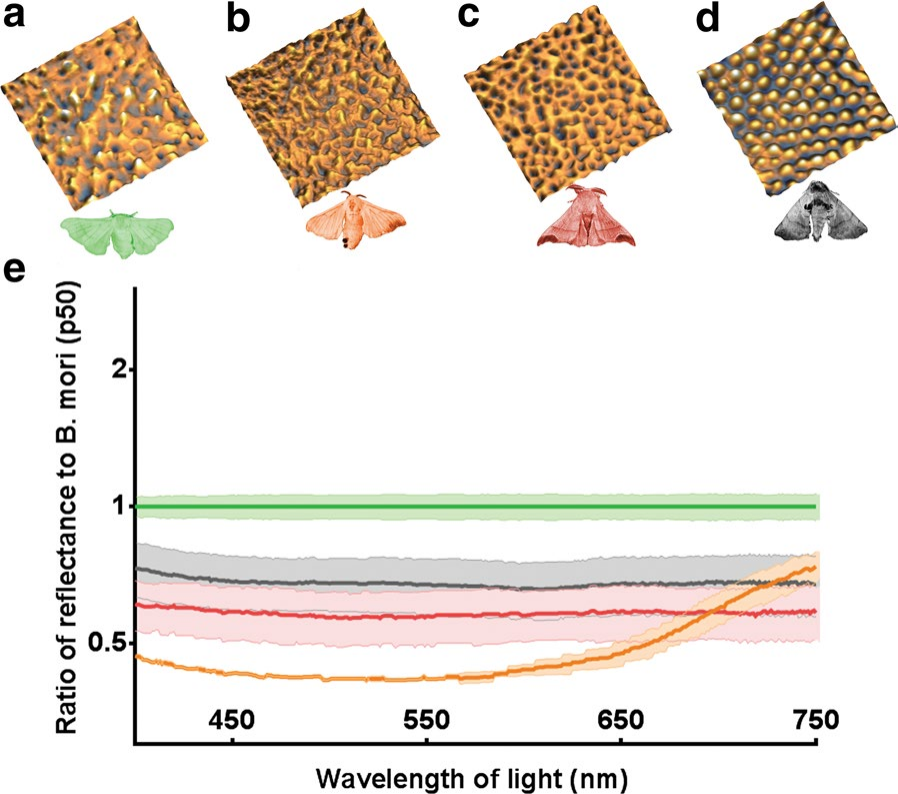
Antireflective function of corneal nanocoatings from *Bombycidae* moths. **a**–**d** 3D AFM representation (3 × 3 µm) of corneal nanocoatings of *B. mori* [Jp] (**a**), *B. mori* [Vn] (**b**), *B. mandarina* (**c**) and *A. torrefacta* (**d**). **e** Ratio of the experimentally measured reflection spectra to the average reflectance of *B. mori* [Jp] measured for *B. mori* [Jp] (*green*), *B. mori* [Vn] (*orange*), *B. mandarina* (*red*) and *A. torrefacta* (*gray*). Data present as mean ± SD, n = 3 (for the experimental data for *B. mori* [Vn] n = 2)

Given these uncertainties, we decided to directly measure reflectivity from corneal surfaces of the *Bombyx* moths. This analysis reveals strong reduction of the reflected light in the broad visible spectrum from the dimpled corneal surfaces of *B. mandarina* and the dimple-to-maze surfaces of *B. mori* [Vn], as compared to the irregularly rough surface of *B. mori* [Jp] (Fig. 2). Remarkably, the antireflective properties provided by the *Bombyx* nano-dimpled coating even exceeded those provided by the nano-pillar arrays of another *Bombycidae* moth, *Apatelodes torrefacta* (Fig. 2d, e). While the nano-dimpled *B. mandarina* arrays (as the nano-pillar *A. torrefacta* arrays) appear to provide uniform broadband anti-reflectivity, the dimple-to-maze *B. mori* [Vn] structures are efficient at low wavelengths and start to decrease anti-reflectivity at >650 nm (Fig. 2e). With the theoretical assessment of these findings currently missing, we are left to speculate that either the lack of uniformity in the overall morphology of the dimple-to-maze nanostructures (Figs. 1e, 2b), or their on average smaller dimensions (Fig. 1g, h) could be the cause of this difference of *B. mori* [Vn] from its wild ancestor. Our findings provide the first demonstration of the antireflective capacity of nano-dimpled surfaces less than 100 nm in depth, indicating that nature has found a relatively low-cost and unpredicted solution to the anti-reflectance.

Corneal nanostructures are built from proteins and lipids placed on the chitin background [26, 27]. The Turing reaction–diffusion model was proposed to govern the formation and diversity of the insect corneal nanostructures [8], which predicts corneal protein(s) to serve as the key slow-diffusing component of this reaction and the building blocks of the resulting structures. To prove directly that corneal proteins are important for formation of the antireflective nanocoatings, we treated corneae of *B. mandarina* with a detergent (see “Materials and methods”), and analyzed the resulting samples for their morphology and anti-reflectance. Remarkably, we find that removal of proteins purges away the nano-dimpled coatings (Additional file 2: Figure S2), correlating with a strong loss of the anti-reflective potential of the corneal surface (Additional file 2: Figure S2b). These features, together with the detailed inspection of the remnant nanostructures’ height and broadness (Additional file 2: Figure S2c, d), indicate that protein extraction brings the surface of *B. mandarina* close to that of *B. mori* [Jp]. These findings prompted us to pinpoint corneal proteins, which may show differential abundance in the wild vs. the domesticated *Bombyx* moths.

**Fig. 3:**
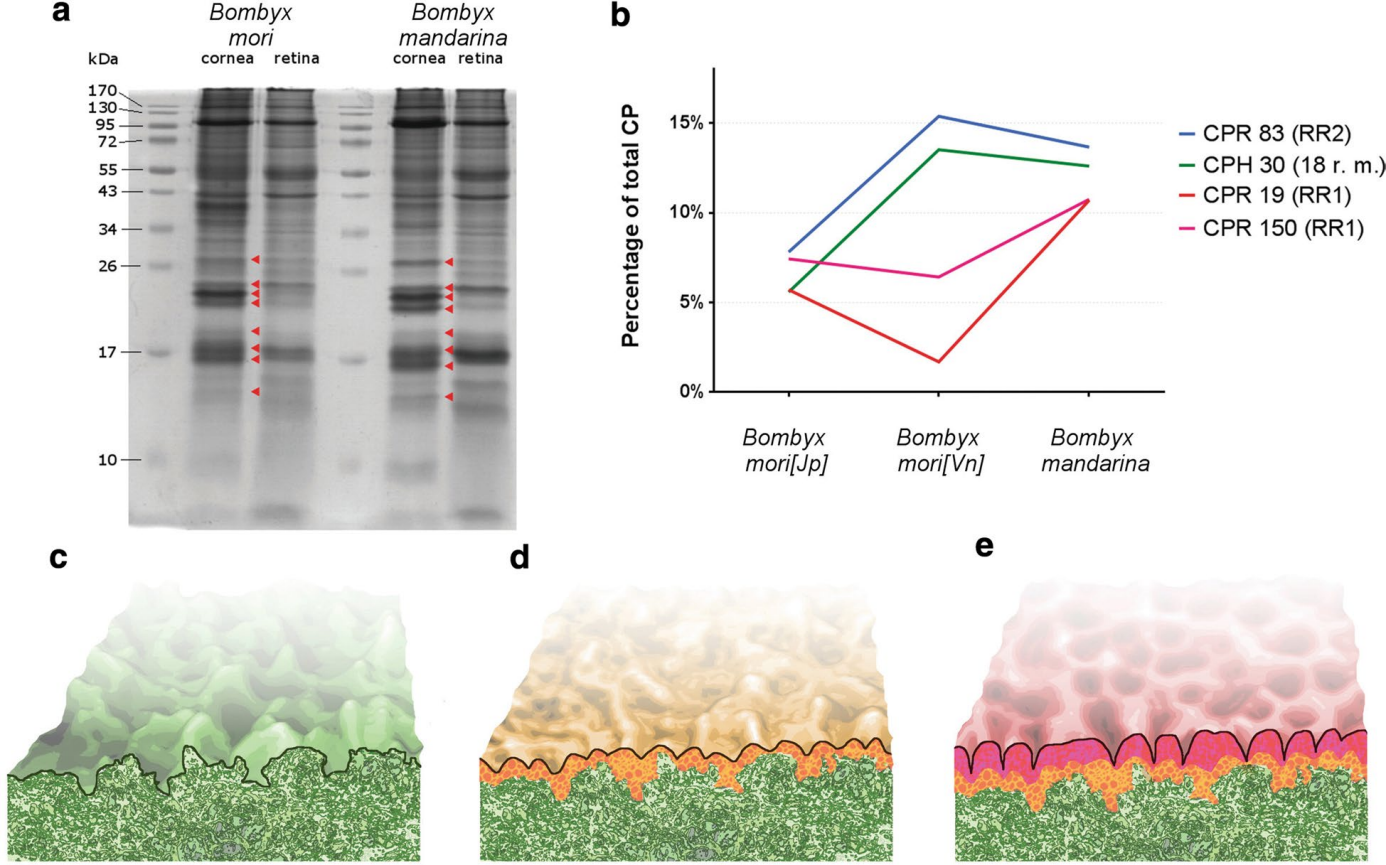
Cuticular proteins in *Bombyx* genus members and the model for nanocoating formation. **a** SDS-PAGE of samples from cornea and retina of *B. mori* [Jp] and *B. mandarina*. Major protein bands unique for cornea (marked by *red arrowheads*) were MS-identified (see Additional file 3: Table S1). **b** The percentage of major cuticular proteins in corneal material from *B. mori* [Jp], *B. mori* [Vn] and *B. mandarina*. **c**–**e** Model of step-wise acquisition of nanostructures on the corneal surface of *B. mori* from Japan (**c**), *B. mori* from Vietnam (**d**), and *B. mandarina* (**e**)

The choice of *B. mori* and *B. mandarina* insects for our analysis was to a large extent dictated by the fact that genomes of these insects have been fully sequenced [18, 19], opening the possibility to identify corneal proteins involved in formation of the nanocoatings—the task not achievable for many other insects (such as *A. torrefacta*) whose genomes have not been sequenced. Thus, we analyzed the protein samples obtained by the detergent extraction (Additional file 2: Figure S2a) of *B. mori* and *B. mandarina*, as well as samples from their underlying retina, by SDS-PAGE followed by mass-spectrometry identification of the most prominent corneal-specific bands (Fig. 3a). This proteomic analysis identified a number of cuticular proteins (CPs, Additional file 3: Table S1), such as CPRs (classical CPs with the chitin-binding domain), and CPHs (hypothetical CPs) [28]. Various chitin-binding domains, RR motifs (where RR2 mediates association with the hard cuticle such as head capsule, and RR1—with soft intermediate membranes), and 18 amino acid repeats can be distinguished in CPs [28].

The major proteins, comprising each >10% and together ca. 50% of the total corneal load in *B. mandarina* corneae, are CPR83, CPR150, CPR19 and CPH30. Remarkably, we find substantial reduction of these proteins in the domesticated moth *B. mori* [Jp] (Fig. 3a, b). CPR83, CPR150, and CPR19 are the regular CPRs containing the chitin-binding domain 4, whereas CPH30 does not contain a chitin-binding domain but has the 18-residue repeats [29]. CPR83 contains an RR2, while CPR150 and CPR19—the RR1 motifs, and thus may mediate interactions with different types of cuticle [28]. Analysis of the corneal proteome of *B. mori* [Vn] revealed similar to *B. mandarina* levels of CPR83 and CPH30, but significantly lower levels of the RR1-family proteins CPR150 and CPR19 (Fig. 3b).

We hypothesize that these changes in the corneal CPs, seen among different *Bombyx* specimen, underlie the differences in the nanostructures that decorate corneae of these strains. Specifically, we propose that the hard-cuticle binding CPR83, possessing the RR2 motif, and the 18-residue repeat-containing CPH30 are the first proteins to be “gained” in *B. mori* [Vn] as compared to *B. mori* [Jp], providing structuring on the corneal surface and strong anti-reflectance (Fig. 3c, d). Next, the soft material-interacting RR1 motif is “recruited” in the form of the CPR19 and CPR150 in corneae of *B. mandarina*, mediating formation of the well-formed nano-dimpled coatings (Fig. 3e). Genetic manipulations (loss-of-function and overexpression) of these proteins in *B. mori* or another insect model is required for the unambiguous testing of our hypothesis, and will be subject of future investigations.

Identification of the candidate proteins governing formation of the *Bombyx* corneal nanocoatings permits creation of novel nanocoatings. Indeed, cloning of the genes encoding for these proteins and their targeted mis- and over-expression in other hosts, such as the genetic model insect *Drosophila melanogaster* [16, 30] is expected to produce novel corneal nanocoatings—potentially with improved physical properties such as anti-reflectance, self-cleaning, anti-bacterial, etc. Together with the similar analysis of the protein composition of corneal nanostructures in other arthropods, this approach opens the door to a high-throughput bioengineering of nanocoatings, which may eventually lead to generation of nanostructures with features interesting for industrial applications.

In regard to the interest for industry, we wish to stress that the nano-dimpled moth-eye nanostructures we have described here display unexpectedly good anti-reflective properties. Given the high cost of industrial generation of the nano-pillar coatings and their sensitivity to physical damage, these nano-dimpled coatings may represent an attractive industrial alternative, given the low cost fabrication of nanohole surfaces [31].

## Materials and methods

The samples of *Bombyx mori* (p50 strain) and *Bombyx mandarina* (Oki line) were provided by the National Bio-Resource Project (NBRP) of the Ministry of Education, Science, Sports and Culture of Japan. Additionally, we studied Vietnamese *B. mori* obtained from the Cuong Hoan Silk Factory, Trung Vuong, Lam Dong Province, Vietnam. The samples of *Apatelodes torrefacta* (mature adults from Louisiana, USA) were obtained from the online shop http://www.thebugmaniac.com.

Preparation and analysis of corneal and retinal samples: Corneal and retinal samples were prepared by cutting off the eyes with a scalpel from the heads of mature adult guillotined *Bombycidae* moths. The retinal material was removed from the immobilized samples into a drop of water by washing and very gentle scrupulous scratching. Upon the separation, the corneal material was further washed 3 times in water. The corneal and retinal samples were extracted from the material of 10 eyes. The samples were boiled for 60 min in the Sample Buffer (62.5 mM Tris–HCl pH 6.8; 10% glycerol; 2% SDS; 1% β-mercaptoethanol; trace of bromophenol blue) prior to separation by 15% SDS-PAGE (Fig. 3) or AFM and reflectance measurement (Additional file 2: Figure S2). Ratio of bands to total protein were counted by the ImageJ software.

Mass-spectrometry (MS): in-gel trypsin digestion and MS were performed by the Protein Analysis Facility of University of Lausanne (Switzerland). The Scaffold viewer software was used for the data analysis with the following parameters: protein threshold 90%, minimum of number of peptides 1, peptide threshold 90%, minimum of probability 90%. The amount of cuticular proteins was counted by using the following formula:

$$\frac{{\mathop \sum \nolimits \left( {\frac{a}{b} \times 100 \times c} \right)_{n} }}{{\mathop \sum \nolimits \left( {\mathop \sum \nolimits \left( {\frac{a}{b} \times 100 \times c} \right)_{n} } \right)_{m} }} \times 100,$$

where *a* is the percentage of total spectra from protein of interest (particular CP) in one band, *b* is the percentage of total spectra of all proteins in one band, *c* is the ratio of band to total protein in the gel, *n* is the number of bands, and *m* is the number of CPs.

The corneal samples of *B. mori* from Vietnam, due to scarceness of material, were directly sent to MS and the results were analyzed as the ratio of the percentage of the total spectra from protein of interest (particular CP) to the percentage of the total spectra of all CPs.

For AFM, corneal samples prepared as described above were attached to a coverslip by a double-sided bonding tape. Microscopy was performed by the NTegra-Prima microscopes (NT-MDT, Zelenograd, Russia) using the contact procedure with the long NSG 11 cantilever (NT-MDT) and the BioScope Resolve, Bruker, with cantilever SCANASYST-AIR. The Gwyddion software [32] was used for visualization and for Fourier analysis.

The same samples were also used for the reflectance measurements, using the JASCO MSV-370 micro-spectrophotometer in the reflection geometry. Using a non-dispersive Schwarzschild-objective and an aperture, the region of interest was set to an area of 300 × 300 µm. The spectral region from beginning of visible spectrum (400 nm) to near infrared (750 nm). The data is used to visualize the spectral ratio $\left( {{{{\text{R}}_{{\left( {\text{of interest}} \right)}} } \mathord{\left/ {\vphantom {{{\text{R}}_{{\left( {\text{of interest}} \right)}} } {{\text{R}}_{{\left( {B. \, mori\,\,\left[ {\text{Jp}} \right]} \right)}} }}} \right. \kern-0pt} {{\text{R}}_{{\left( {B. \, mori\,\,\left[ {\text{Jp}} \right]} \right)}} }}} \right)$ between the two species.

## Additional files

**Additional file 1: Figure S1.** Fourier analysis of the *Bombyx* corneal nanostructures. **a–f**, Example of structures with increasing degree of disordering (artificial sets for panels **a** to **c**: panel size 1.3 µm, height 30 nm) and their Fourier analysis (**d–f**). **g–l**, AFM scans (**g**–**i**) of corneal surfaces of different *Bombyx* samples (**g**, *B. mandarina*, **h**, *B. mori* [Vn], **i**, *B. mori*[Jp]) and the corresponding Fourier spectra (**j**–**l**). This analysis reveals quasi-random vs. random structures in different samples. Quasi-random structures retain a stable period between any two neighboring peaks resulting in a ring of reflexes in their Fourier analysis (**a**, **d**). Alternatively, quasi-random structures can lack a stable period but fill the space uniformly, producing a large lattice covered by reflexes on the Fourier spectrum (**b**, **e**). Instead, random structures fill the space lopsidedly and despite the fact that the ratio of large and small objects and distances between them may exactly match those of the quasi-random structures, the Fourier spectrum shows a significant decrease of the lattice size (**c**, **f**). Fourier analysis of the *B. mori* [Jp] corneal nanocoatings shows that the size of lattice (**i**, **l**) of reflexes is just 12 μm^−1^, significantly smaller than in the case of those *B. mori* [Vn] and *B. mandarina* (17 μm^−1^, **g**, **h**, **j**, **k**). In the absence of clearly defined reflections, these two last-mentioned structures could be recognized as the quasi-random dimpled patterns, while the lattice size of Fourier spectrum of *B. mori* [Jp] indicates absence of any ordering.

**Additional file 2: Figure S2.** Detergent treatment removes nanostructures and anti-reflectivity in *B. mandarina* corneae. **a,** Detergent treatment purges away the nano-dimpled pattern of *B. mandarina* corneae. Images pre- and post-treatment are 3.5 × 3.5 µm. The height dimension of the surface (in nm) is indicated by the color scale at the right panel with the mean set to zero. **b**, Ratio of the experimentally measured reflection spectra to the average reflectance of *B. mori*[Jp] measured for *B. mori*[Jp] (green), *B. mandarina* (red) and detergent-treated *B. mandarina* (blue). Data present as mean ± SD, n = 3. **c**,** d**, Calculation of the height of protrusions (from the lowest point up to the next highest point, **c**) and their broadness (**d**) of *B. mori* [Jp] (in green), *B. mandarina* (in red), and detergent-treated *B. mandarina* (in blue); n = 50.

**Additional file 3: Table S1.** Ratio of individual CPs to total amount of CPs.

## Abbreviations

AFM: atomic-force microscopy
CPs: cuticular proteins
MS: mass-spectrometry
