# Supplementary figures and images for: Alternative moth-eye nanostructures: antireflective properties and composition of dimpled corneal nanocoatings in silk-moth ancestors
Source: J Nanobiotechnology. 2017 Sep 6;15:61. doi: 10.1186/s12951-017-0297-y (PMC5588701; doi:10.1186/s12951-017-0297-y)

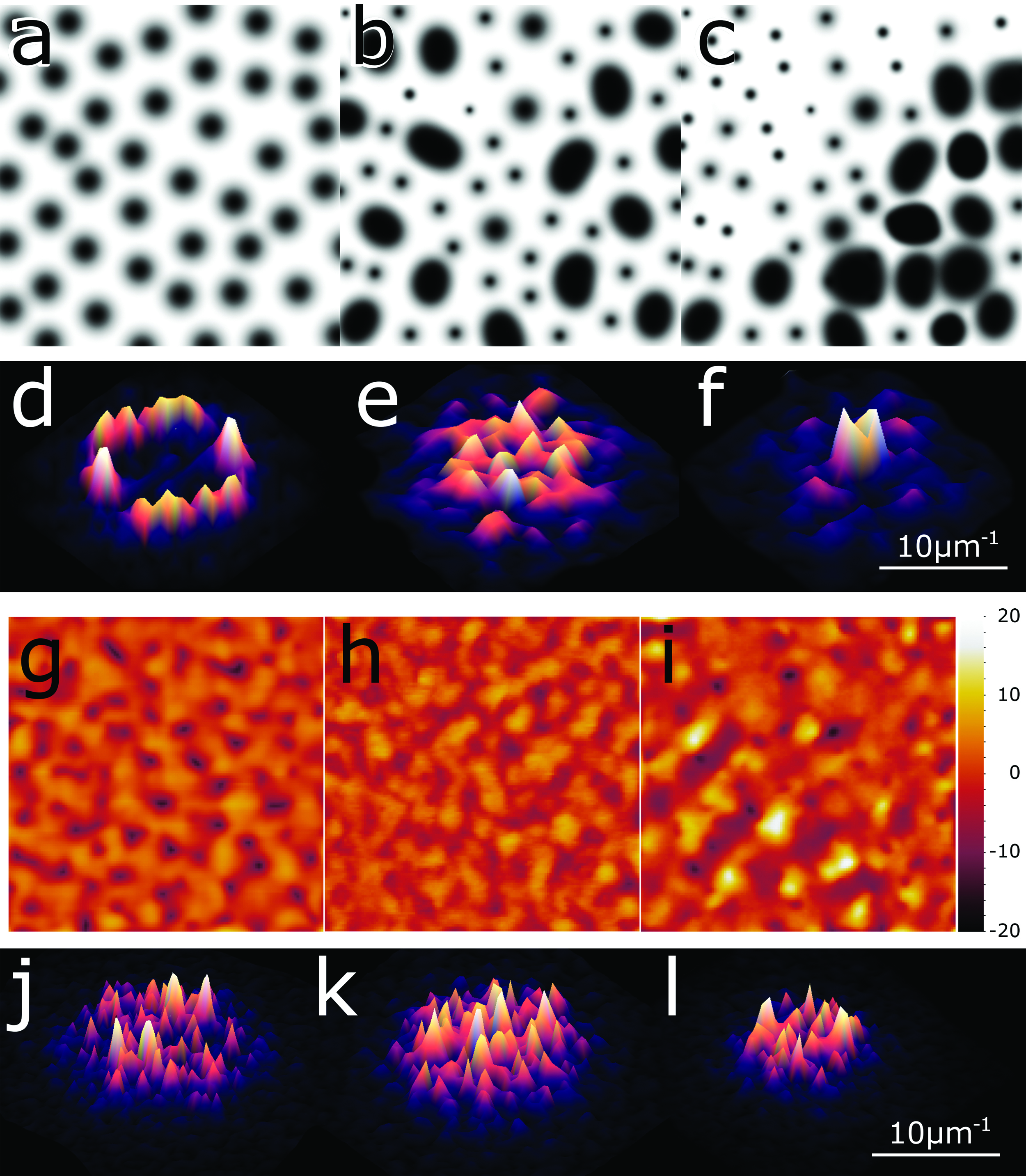

Supplement: Supplementary file 1 — Additional file 1: Figure S1. Fourier analysis of the Bombyx corneal nanostructures. a–f, Example of structures with increasing degree of disordering (artificial sets for panels a to c: panel size 1.3 µm, height 30 nm) and their Fourier analysis (d–f). g–l, AFM scans (g–i) of corneal surfaces of different Bombyx samples (g, B. mandarina, h, B. mori [Vn], i, B. mori[Jp]) and the corresponding Fourier spectra (j–l). This analysis reveals quasi-random vs. random structures in different samples. Quasi-random structures retain a stable period between any two neighboring peaks resulting in a ring of reflexes in their Fourier analysis (a, d). Alternatively, quasi-random structures can lack a stable period but fill the space uniformly, producing a large lattice covered by reflexes on the Fourier spectrum (b, e). Instead, random structures fill the space lopsidedly and despite the fact that the ratio of large and small objects and distances between them may exactly match those of the quasi-random structures, the Fourier spectrum shows a significant decrease of the lattice size (c, f). Fourier analysis of the B. mori [Jp] corneal nanocoatings shows that the size of lattice (i, l) of reflexes is just 12 μm−1, significantly smaller than in the case of those B. mori [Vn] and B. mandarina (17 μm−1, g, h, j, k). In the absence of clearly defined reflections, these two last-mentioned structures could be recognized as the quasi-random dimpled patterns, while the lattice size of Fourier spectrum of B. mori [Jp] indicates absence of any ordering. [file 12951_2017_297_MOESM1_ESM.jpg]

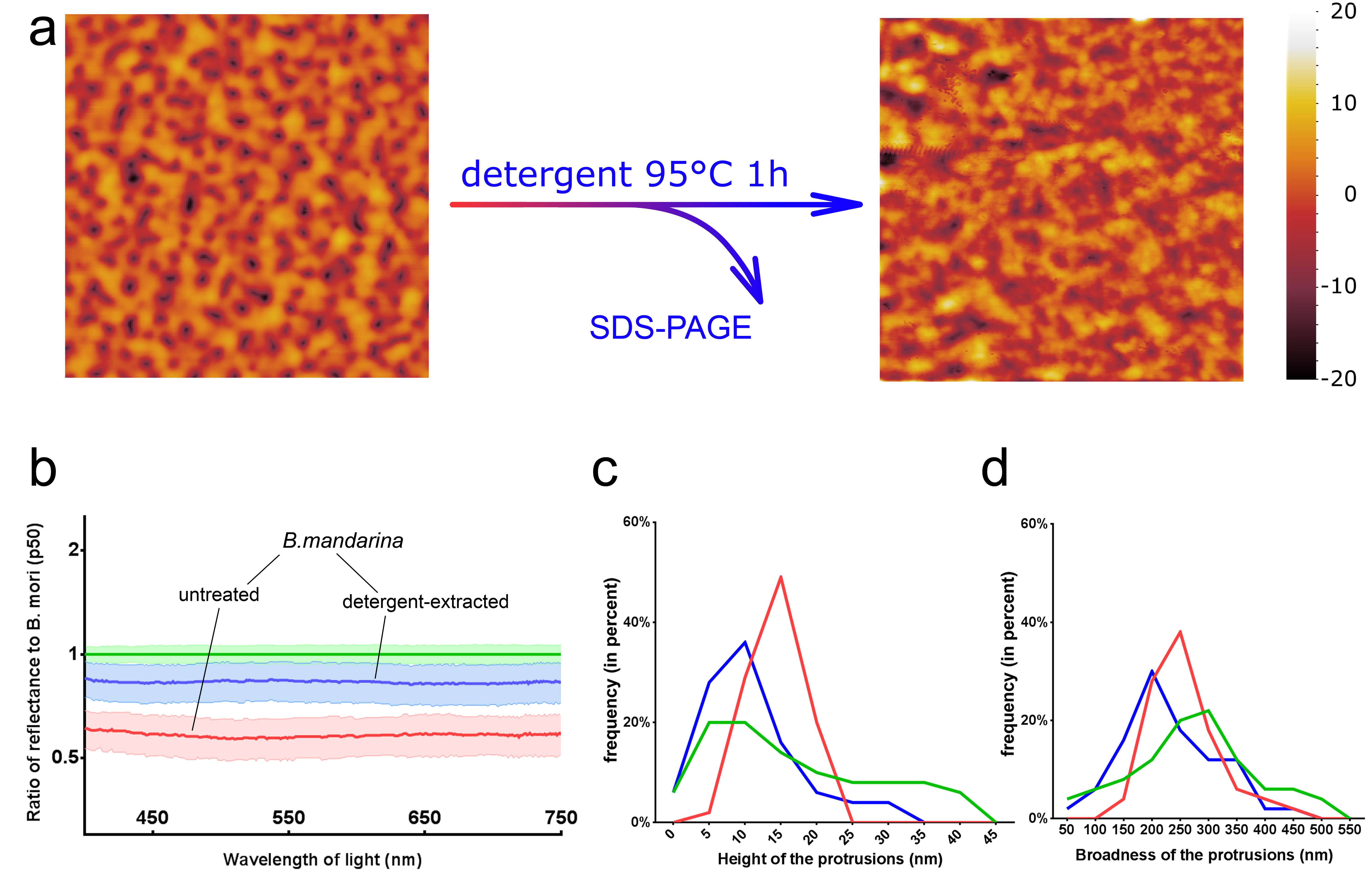

Supplement: Supplementary file 2 — Additional file 2: Figure S2. Detergent treatment removes nanostructures and anti-reflectivity in B. mandarina corneae. a, Detergent treatment purges away the nano-dimpled pattern of B. mandarina corneae. Images pre- and post-treatment are 3.5 × 3.5 µm. The height dimension of the surface (in nm) is indicated by the color scale at the right panel with the mean set to zero. b, Ratio of the experimentally measured reflection spectra to the average reflectance of B. mori[Jp] measured for B. mori[Jp] (green), B. mandarina (red) and detergent-treated B. mandarina (blue). Data present as mean ± SD, n = 3. c, d, Calculation of the height of protrusions (from the lowest point up to the next highest point, c) and their broadness (d) of B. mori [Jp] (in green), B. mandarina (in red), and detergent-treated B. mandarina (in blue); n = 50. [file 12951_2017_297_MOESM2_ESM.jpg]
