# Supplementary material for: Alternative moth-eye nanostructures: antireflective properties and composition of dimpled corneal nanocoatings in silk-moth ancestors
Source: J Nanobiotechnology. 2017 Sep 6;15:61. doi: 10.1186/s12951-017-0297-y (PMC5588701; doi:10.1186/s12951-017-0297-y)
Supplement: Supplementary file 3 — Additional file 3: Table S1. Ratio of individual CPs to total amount of CPs. [file 12951_2017_297_MOESM3_ESM.docx]

**Supplementary Table 1**: Ratio of individual CPs to total amount of CPs

| Protein | *Bombyx mandarina* | *Bombyx mori* [Vn] | *Bombyx mori* [Jp] |
| --- | --- | --- | --- |
| CPR83 | 0.136655238 | 0.153779 | 0.078198 |
| CPH30 | 0.126013845 | 0.13519 | 0.055844 |
| CPR150 | 0.107562423 | 0.064215 | 0.074335 |
| CPR19 | 0.10699337 | 0.016899 | 0.057111 |
| CPR87 | 0.079155889 | 0.109842 | 0.069141 |
| CPR65 | 0.036915463 | 0.054076 | 0.047526 |
| CPH4 | 0.026737747 | 0.059146 | 0.031233 |
| CPH2 | 0.0267447 | 0.047316 | 0.0414 |
| CPR73 | 0.015531479 | 0.001859 | 0.066384 |
| CPFL1 | 0.019155342 | 0.047316 | 0.014445 |
| CPH31 | 0.020875311 | 0.01504 | 0.036191 |
| CPR56 | 0.025138715 | 0.002366 | 0.038373 |
| CPH28 | 0.014828461 | 0.023658 | 0.019846 |
| CPR75 | 0.007307434 | 0.009294 | 0.038699 |
| CPR92 | 0.00392032 | 0.040557 | 0.008188 |
| CPR10 | 0.011943341 | 0.018589 | 0.019766 |
| CPR67 | 0.000735312 | 0 | 0.045869 |
| CPG13 | 0.003434424 | 0.037177 | 0.004582 |
| CPH5 | 0.010244691 | 0.007097 | 0.025363 |
| CPR74 | 0.014990085 | 0.002366 | 0.02391 |
| CPR69 | 0.021517475 | 0.002535 | 0.015098 |
| CPR93 | 0 | 0.038867 | 0 |
| CPR84 | 0.029782244 | 0 | 0.003241 |
| CPH42 | 0.006563907 | 0.011998 | 0.01438 |
| CPH1 | 0.011579621 | 0.007604 | 0.011659 |
| CPR91 | 0 | 0 | 0.030407 |
| CPR135 | 0.021653635 | 0 | 0.008001 |
| CPH8 | 0.009308504 | 0.016223 | 0.002762 |
| CPR71 | 0.001891487 | 0.004056 | 0.020271 |
| CPR57 | 0.011370718 | 0.006928 | 0.003521 |
| CPR78 | 0.006939698 | 0.006928 | 0.006365 |
| CPR37 | 0.002639382 | 0 | 0.016334 |
| CPH9 | 0.013619427 | 0 | 0 |
| CPR54 | 0.001035968 | 0.008618 | 0.003505 |
| CPR72 | 0.005770369 | 0.000845 | 0.005946 |
| CPR63 | 0.000516212 | 0.001267 | 0.010108 |
| CPR17 | 0.010080556 | 0 | 0.001501 |
| CPR55 | 0.007817422 | 0.001048 | 0.002311 |
| CPG31 | 0.000735312 | 0.000422 | 0.009773 |
| CPR34 | 0.005032083 | 0.000625 | 0.00449 |
| CPH17 | 0.004481098 | 0.001267 | 0.003993 |
| CPG12 | 0 | 0.009632 | 0 |
| CPG38 | 0.000420862 | 0.004901 | 0.00168 |
| CPR136 | 0.004938376 | 0 | 0.001918 |
| CPR15 | 0.000937074 | 0.002873 | 0.002795 |
| CPR48 | 0.000420862 | 0.005408 | 0 |
| CPH20 | 0.003467752 | 0.000422 | 0.001918 |
| CPR79 | 0.000255695 | 0 | 0.005139 |
| CPR68 | 0.002600507 | 0.002535 | 0 |
| CPR52 | 0 | 0.000203 | 0.003687 |
| CPH21 | 0.002442828 | 0 | 0.001421 |
| CPR77 | 0 | 0 | 0.003687 |
| CPG39 | 0 | 0.002366 | 0.001095 |
| CPR33 | 0.002969503 | 0 | 0 |
| CPR59 | 0.001926858 | 0 | 0.000597 |
| CPR70 | 0 | 0.001048 | 0.001321 |
| CPG26 | 0.000481403 | 0.001859 | 0 |
| CPR95 | 0.000615106 | 0.001673 | 0 |
| CPR101 | 0.000420862 | 0.001859 | 0 |
| CPH3 | 0.000420862 | 0.001673 | 0 |
| CPG15 | 0.000240701 | 0.000845 | 0.000948 |
| CPR66 | 0 | 0.002028 | 0 |
| CPR103 | 0 | 0 | 0.001843 |
| CPG24 | 0 | 0.00147 | 0 |
| CPR126 | 0.001466447 | 0 | 0 |
| CPR148 | 0.001459161 | 0 | 0 |
| CPG14 | 0 | 0.000625 | 0.000597 |
| CPH36 | 0.000774833 | 0.000422 | 0 |
| CPH11 | 0.000735312 | 0.000422 | 0 |
| CPR80 | 0.001047387 | 0 | 0 |
| CPR94 | 0.000942949 | 0 | 0 |
| CPH15 | 0.000927955 | 0 | 0 |
| CPR128 | 0 | 0.000845 | 0 |
| CPR129 | 0.000774833 | 0 | 0 |
| CPR82 | 0.000687254 | 0 | 0 |
| CPG3 | 0.000661564 | 0 | 0 |
| CPG41 | 0 | 0.000625 | 0 |
| CPR149 | 0 | 0 | 0.000625 |
| CPG20 | 0.000503641 | 0 | 0 |
| CPR51 | 0 | 0 | 0.000474 |
| CPH41 | 0 | 0.000422 | 0 |
| CPR122 | 0 | 0.000422 | 0 |
| CPR151 | 0 | 0.000203 | 0.000184 |
| CPR64 | 0.000240701 | 0 | 0 |
| CPH32 | 0 | 0.000203 | 0 |
